# Ultrasonographic evaluation of nail matrix topography for preservative nail surgery of nail unit melanoma

**DOI:** 10.1111/1346-8138.17340

**Published:** 2024-06-14

**Authors:** Gi Ung Ha, Jin Ho Kim, Dae‐Lyong Ha, Hoseok Lee, Seok‐Jong Lee

**Affiliations:** ^1^ Department of Dermatology, School of Medicine Kyungpook National University Daegu Korea; ^2^ Semyung Radiologic Clinic Gumi Korea

**Keywords:** functional surgery, melanoma, nail unit melanoma, ultrasonography

## Abstract

Recently, functional or preservative surgery has been preferred for nail unit melanoma; however, complete resection of the nail unit, particularly the matrix, is challenging because of its complex structure. This study aims to measure the distance of important nail structures through ultrasonography. Herein, 14 patients without nail deformity were included. The length from the nail cuticle to the distal interphalangeal joint (distance X), to the attachment part of the extensor muscle (distance A), to the median proximal end of the nail matrix (distance B), and to the lateral proximal end of the nail matrix (distance C) were measured. In the axial plane, the length from the highest point of the nail plate to the bottom of the distal phalanx (distance Y) and to the lateral tip of the nail plate (distance D) were measured. On the first fingernail, third fingernail, first toenail, and third toenail, the mean ratio A:X, ratio B:X, ratio C:X, and ratio D:Y were 78.6%, 44.3%, 57.2%, 40.1%, and 84.6%; 55.9%, 64.9%, 40.2%, and 66.4%; 35.6%, 50.8%, 34.3%, and 81.9%; and 57.2%, 59.6%, and 31.7%, respectively. Nail units are often invisible to the naked eye; thus, this study will help identify the approximate scope of excision.

## 1 BACKGROUND

A nail unit melanoma is a skin malignancy arising from the nail matrix and other perinail tissues. It is common among patients aged 50 to 70 years, with a predilection for the thumb and hallux.^1^ Although it accounts for only approximately 3% of all melanomas in White individuals, it accounts for approximately 18% of all melanomas in Korean individuals.^2^, ^3^ In recent years, digit‐sparing approaches such as functional surgery or preservative surgery as a surgical intervention are preferred rather than digit amputation if possible. Complete removal of the nail units, including the nail matrix, is crucial; however, achieving a complete resection is challenging owing to the complicated three‐dimensional structure of the nail unit and the fact that the nail matrix range cannot be recognized with the naked eye.^4^ Therefore, if a complete excision is not performed, the melanoma will recur and nail fragments will grow back from the remaining nail matrix, causing pain.^5^ Conversely, when excessive resection is attempted to prevent such complications, there is a risk of damaging the attachments between the tendons and bones where the extensor muscle of the digits is inserted, which are responsible for the extensor movement of the fingers and toes. If these tendons are damaged, permanent flexion contracture of the joint of the operated finger or toe can occur.^6^ Therefore, surgeons need to be adept of the complex anatomy and topography of the area, including its relationship with the surrounding structures. They require knowledge of the central distance between the matrix and the distal interphalangeal joint (DIPjt) crease to avoid damaging the tendon insertion. Furthermore, knowing the position of the proximal lateral tips of the nail matrix and the lateral depth of the nail plate is necessary. However, due to the insufficient knowledge regarding these structures, an ultrasound was used to conduct this study.

## 2 METHODS

**FIGURE 1:**
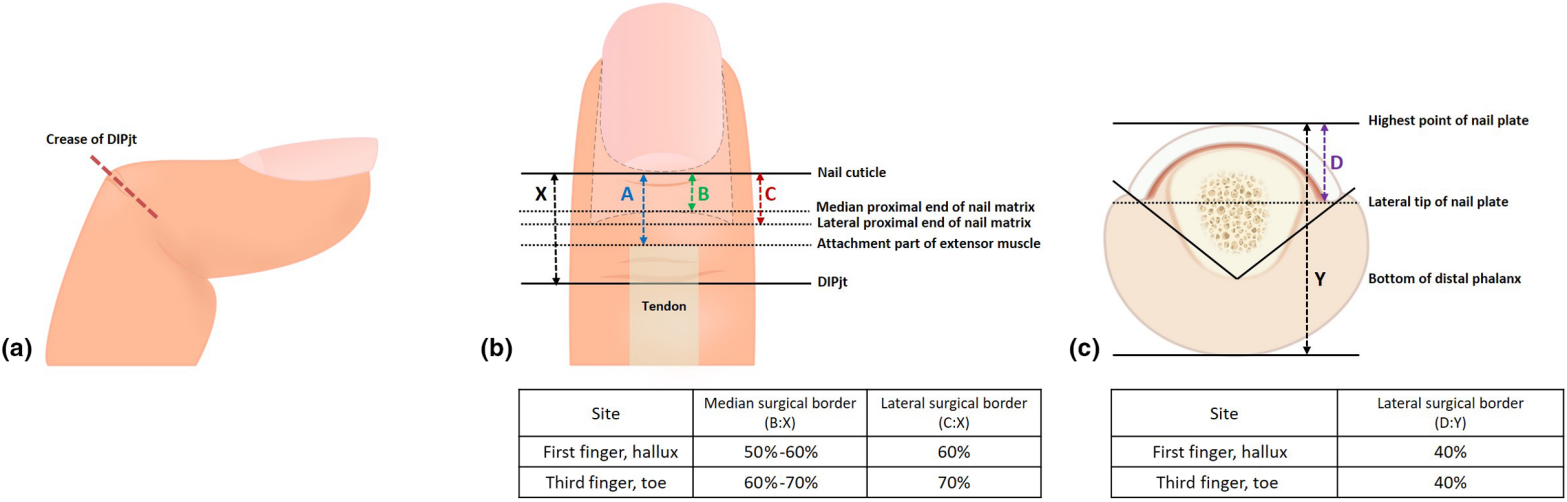
(a) A transverse line drawn along the visible skin crease is shown reflecting the distal interphalangeal joint (DIPjt) when maximally flexed. (b) A schematic diagram of important structures in functional surgery. X, length from the nail cuticle to the DIPjt; a, length from the nail cuticle to the attachment part of the extensor muscle; b, length from the nail cuticle to the median proximal end of the nail matrix; c, length from the nail cuticle to the lateral proximal end of the nail matrix. The approximate median and lateral surgical border of each site is summarized in the Table. (c) A axial schematic diagram of the nail. Y, length from the highest point of the nail plate to the bottom of the distal phalanx; d, length from the highest point of the nail plate to the lateral tip of the nail plate. The approximate lateral surgical border of each site summarized in the Table.

A total of 14 patients who underwent functional surgery for nail unit melanoma at the Department of Dermatology, Kyungpook National University Hospital, from September 2020 to March 2021, and those who did not have nail deformity due to trauma on the other hand and foot region or other nail diseases were included in the study. Ultrasound was performed on the first and third fingernails and first and third toenails of the opposite side that underwent finger/toe surgery. Using a Philips EpiQ‐7 device, ultrasound measurements were conducted by a radiologist (H.L.) under the supervision of a dermatologist (S.L.). This study was approved by the institutional review board (IRB) of Kyungpook National University Hospital (IRB number: KNUH 2020–07–026‐001). The outline of this study is presented in Figure 1. A line was drawn transversely along the visible skin crease reflecting the DIPjt when it was maximally flexed (Figure 1a), and an ultrasound was performed on the first and third fingernails/toenails of the patients. The nail cuticle length from the skin crease line marking the maximum flexion edge of the DIPjt (distance X), to the attachment part of the extensor muscle (distance A), to the median proximal end of the nail matrix (distance B), and to the lateral proximal end of the nail matrix (distance C) were measured, respectively (Figure 1b). In the axial plane, the length from the highest point of the nail plate to the bottom of the distal phalanx (distance Y) and to the lateral tip of the nail plate (distance D) were measured (Figure 1c). Since the size of each person's finger is different, these measured values are summarized as a percentage for each length, not the length itself (Table 1).

**TABLE 1:** Percentage of the length of A, B, C, D, X, and Y from a sonographic analysis of the nail.

Parameter (%)	First finger	Third finger	First toe	Third toe
A:X (tendon distance)	78.6 ± 4.3	84.6 ± 5.0	66.4 ± 5.6	81.9 ± 3.6
B:X (median proximal matrix distance)	44.3 ± 4.9	55.9 ± 7.8	35.6 ± 6.6	57.2 ± 15.1
C:X (lateral proximal matrix distance)	57.2 ± 4.5	64.9 ± 5.3	50.8 ± 9.7	59.6 ± 11.5
D:Y (lateral plate extension)	40.1 ± 5.4	40.2 ± 6.2	34.3 ± 3.9	31.7 ± 4.9

*Note*: Values are presented as mean ± standard deviation.

## 3 RESULTS

### 3.1 Longitudinal length of the nail unit

On the first fingernails, the mean ratio A:X (tendon distance), ratio B:X (median proximal matrix distance), and ratio C:X (lateral proximal matrix distance) was 78.6%, 44.3%, and 57.2%, respectively. On the third fingernails, the mean ratio A:X, ratio B:X, and ratio C:X was 84.6%, 55.9%, and 64.9%, respectively. On the first toenails, the mean ratio A:X, ratio B:X, and ratio C:X was 66.4%, 35.6%, and 50.8%, respectively. On the third toenails, the mean ratio A:X, ratio B:X, and ratio C:X was 81.9%, 57.2%, and 59.6%, respectively. In terms of percentage of length, the first fingernail was similar to the first toenail and the third fingernail was similar to the third toenail.

### 3.2 Sagittal depth of the nail unit

On the first and third fingernails, the mean ratio D:Y (lateral plate extension) was 40.1% and 40.2%, respectively. On the first and third toenails, the mean ratio D:Y was 34.3% and 31.7%, respectively. The first fingernail was similar to the first toenail and the third fingernail was similar to the third toenail in percentage of depth.

## 4 DISCUSSION

This study aimed to measure the distance of important structures of digit‐sparing functional surgery via ultrasonography. It was conducted aiming to achieve a complete resection of the whole nail plate with melanoma, prevent flexion contracture by sparing some structures such as the extensor tendon, and further suggest safety margins for functional surgery. Amputation may be more optimal for a clear excision of the nail unit melanoma; however, for cosmetic and functional purposes, digit‐sparing approaches such as functional surgery is preferred, unless absolutely necessary. However, because nails have a complex three‐dimensional structure, limited literature exists regarding the extent of the resection margin and whether Mohs surgery is helpful or not.^7^ According to the results of this study, when performing functional surgery on the thumb and hallux, to definitely remove the nail matrix, the median surgical border should be approximately 50% to 60% and the lateral surgical border should be approximately 60% or more of the length from the nail cuticle to the DIPjt. In the axial plane, the lateral surgical border should be approximately 40% or more of the length from the highest point of the nail plate to the bottom of the distal phalanx. On the fingernails/toenails other than the thumb and hallux, the median and lateral surgical borders should be approximately 60% to 70% or 70% or more of the length from the cuticle to the DIPjt, respectively. The lateral surgical border of the axial plane should be approximately 40% or more of the length from the highest point of the nail plate to the bottom of the distal phalanx. Based on the above anatomical structure, the whole nail plate with melanoma in all enrolled patients completely resected and confirmed it microscopically by both dermatologist and pathologist. There was no patient whose tendon was damaged.

Therefore, the operator's experience in nail unit surgery and continuous evaluation of the remaining lesion is important in functional surgery. However, nail units are often invisible to the naked eye; therefore, this study will be helpful in determining the approximate scope of excision.

## CONFLICT OF INTEREST STATEMENT

The authors declare no conflicts of interest related to this article.
